# Elevated intracellular chloride level in albino visual cortex neurons is mediated by Na-K-Cl co-transporter

**DOI:** 10.1186/1471-2202-9-57

**Published:** 2008-06-30

**Authors:** Dmitry Diykov, Andrey Turchinovich, Georg Zoidl, Klaus-Peter Hoffmann

**Affiliations:** 1 International Graduate School of Neuroscience, Ruhr University Bochum, FNO 01/114 Universitätsstr.150, 44801, Bochum, Germany; 2 Department of Neuroanatomy & Medicine, MA 6 Ruhr-University Bochum, D-44780 Bochum, Germany; 3 Department of General Zoology & Neurobiology, Ruhr-University Bochum, D-44780 Bochum, Germany

## Abstract

**Background:**

During development the switch from a depolarizing to a hyperpolarizing action of GABA is a consequence of a decrease of the Na^+^-K^+^-2Cl^- ^co-transporter (NKCC1, Cl^-^-uptake) and increase of the K^+^-Cl^- ^co-transporter (KCC2, Cl^-^-extrusion) expression. However albino visual cortex neurons don't show a corresponding decrease in intracellular chloride concentration during development of the visual system as compared to pigmented animals.

**Results:**

Our study revealed that more cells express NKCC1 in albinos compared to pigmented rat visual cortex neurons whereas KCC2 is expressed in all cells in both strains. We determined a positive relationship between the presence of NKCC1 and an inhibitory deficit in single neurons of the albino visual cortex. After pharmacological blockade of NKCC1 function with its specific inhibitor, bumetanide, the reversal potential of electrically evoked GABAA receptor-mediated postsynaptic currents and, as a consequence, [Cl-]_i _in albino visual cortex neurons shifted to the pigmented rat brain value. In conclusion, our pharmacological experiments and subsequent single cell real time PCR analysis of the co-transporter mRNA demonstrated that the inhibitory deficit present in the albino visual cortical network is almost exclusively mediated by NKCC1.

**Conclusion:**

Our findings suggest that blocking of NKCC1 in albino visual cortex neurons could improve processing in visual cortex and therefore might be beneficial for vision in albinos.

## Background

Albino mutations lead to a decrease in direction selectivity of neurons in the motion sensitive cortical and subcortical areas of albino mammals [1,2]. This, in turn critically depends on GABAergic mechanisms [3-6]. While GABA is the main inhibitory transmitter in the adult brain, GABAergic transmission is excitatory during early postnatal development. This different action of GABA results from a reversed chloride concentration gradient with higher intracellular chloride concentration in immature neurons [7-10]. The Na^+^-K^+^-2Cl^- ^co-transporter (NKCC1, Cl^-^-uptake) and the K^+^-Cl^- ^co-transporter (KCC2, Cl^-^-extrusion) are the most important of the many known chloride regulators in neocortical neurons [11,12]. The developmental switch to an inhibitory action of GABA is a consequence of a decrease of NKCC1 and increase of KCC2 expression after birth. Cl^- ^uptake in immature neurons is mediated by Na^+^-K^+^-2Cl^- ^cotransporter [8,10,13], as no GABA_A _mediated depolarization was found in NKCC1 knock out mice [14]. Interestingly in cortical neurons, a shift in Cl- homeostasis toward a higher [Cl-]_i _is implicated in the determination of developmental stage: depolarizing GABAergic and glycinergic responses mediate various developmental processes, such as neuronal migration, differentiation, and synapse formation [7,15-17]. In contrast to NKCC1, the KCC2 co-transporter's central role is promoting inhibition and preventing hyperexcitability [9,18]. An inhibitory action of GABA is required to discriminate differences in input activities during processes accompanied by synaptic pruning [19], i.e. the presence of GABAA receptor-mediated inhibition is essential during the critical period of ocular-dominance plasticity [20]. Zhu et al. showed in KCC2^-/- ^mice [21] that cortical neurons lacking KCC2 not only fail to show a developmental decrease in [Cl^-^]_i_, but also are unable to regulate [Cl^-^]_i _on Cl^- ^loading or maintain [Cl]_i _during membrane depolarization.

## Results and discussion

To test whether changed GABA_A_R mediated currents observed in albino visual cortex neurons [22] is regulated by the two major cation-chloride co-transporters: KCC2 and NKCC1, the mRNAs for these transporters were studied in the albino and pigmented visual cortex neurons of postnatal day (P) 20–40 rats by single cell real time PCR. Out of 61 neurons tested in both groups (31 for albino and 30 for pigmented), 27 were found to express NKCC1. Of these, 22 neurons were from albino and 5 from pigmented rat visual cortex. (p < 0,001, Chi-square test, data summarized in table 1). This in contrast to Yamada et al. who using semiquantitative single cell multiplex RT PCR didn't find any NKCC1 mRNA in neurons of the somatosensory cortex in P11-20 albino (Wistar) rats [10]. KCC2 mRNA was detected in every cell tested in both groups (pigmented and albinos). Accordingly, mRNA expression of the house-keeping gene β-actin was also found in every cell analyzed and served as internal control for PCR efficiency. This fact may argue that regulation of the major outward chloride co-transporter is not impaired, or not impaired to such an extent as its inward counterpart. As our two-steps real time single cell PCR did not reveal a qualitative difference in KCC2 mRNA expression and because no reliable co-transporter blocker for KCC2 is available [18], further investigations are needed to quantify the co-transporter participation in intracellular chloride regulation in albino visual cortex neurons compared to the brain of pigmented animals. Interestingly, we detected several NKCC1 positive neurons in P20-40 pigmented rat visual cortex in agreement with the evidence for NKCC1 in adult human neocortex [23] and in rat visual cortex [19] received by use of other methods.

**Table 1:** NKCC1 positive and negative cells in albino and pigmented rat visual cortex

NKCC1 mRNA	+	-	n
Albino neurons	22*	9	31
Pigmented neurons	5	25	30

Distribution of NKCC1 positive and negative cells as revealed by single cell real time PCR in albino and pigmented rat visual cortex (*p < 0.001, Chi-square test).

**Figure 1:**
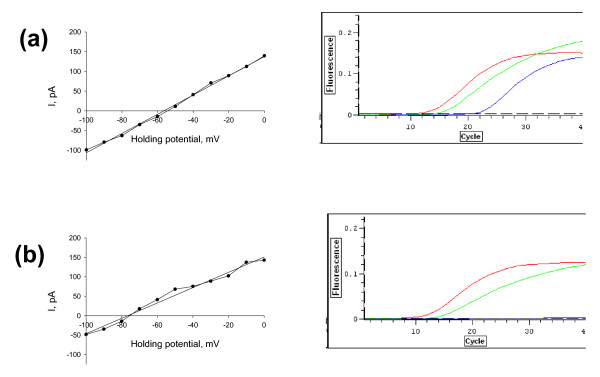
**Sample current-voltage curves of the electrically evoked GABAAR-mediated postsynaptic currents and fluorescence curves of tested PCR products**. Sample current-voltage curves of the electrically evoked GABAAR-mediated postsynaptic currents and fluorescence curves of tested PCR products (red well for β-actin, green for KCC2 and blue for NKCC1) in NKCC1 positive (a) and NKCC1 negative visual cortex neurons (b). Note the E_GABA _shift in positive direction in NKCC1 positive neurons.

**Figure 2:**
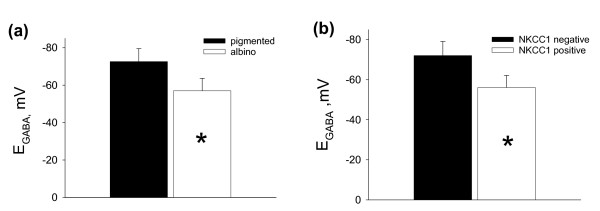
**Differences in E_GABA _between albino and pigmented rat visual cortex neurons and between NKCC1 positive and negative cells**. Differences in the reversal potential of GABAAR-mediated postsynaptic currents between albino and pigmented rat visual cortex neurons (a) and NKCC1 positive and negative cells (b). Data are presented as mean ± S.D (*p < 0.001, Mann-Whitney rank sum test).

**Figure 3:**
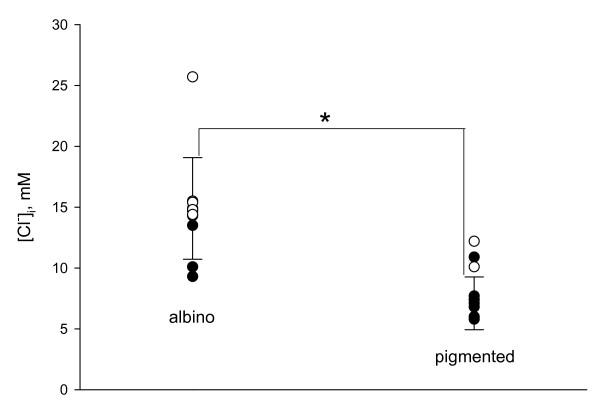
**Relationship between [Cl^-^]_i _level and expression of NKCC1 co-transporter in albino and pigmented rat visual cortex neurons**. Relationship between [Cl^-^]_i _level and expression of NKCC1 co-transporter in albino and pigmented rat visual cortex neurons. Open circles represent NKCC1 positive cells, full circles – negative cells. Data are presented as mean ± S.D (*p < 0.001, Mann-Whitney rank sum test).

In order to directly correlate Cl^- ^transporter RNA expression to electrophysiology, we performed gramicidin-perforated patch clamp recordings and subsequent analysis of NKCC1 mRNA expression in the cytoplasm of the measured cells. Current-voltage curves of the electrically evoked GABAAR-mediated postsynaptic currents in NKCC1 positive (a) and NKCC1 negative neurons (b) as well as the corresponding sample fluorescence curves of PCR products are presented in fig. 1. We found a statistically significant difference in the reversal potential of GABAAR-mediated postsynaptic currents (E_GABA_) between albino and pigmented rat visual cortical neurons (fig. 2a): -57 +/- 6.6 mV (n = 11) and -72.6 +/- 6.9 mV (n = 10, p < 0.001, Mann-Whitney rank sum test) respectively. The E_GABA _value obtained for NKCC1 expressing cells was -56.1 +/- 6 mV (n = 10), while for NKCC1 negative neurons this value comprised -72 +/- 7.1 mV (n = 11, p < 0,001, Mann-Whitney rank sum test) (fig. 2b). Additionally, the presence of NKCC1 RNA strongly correlated with high intracellular chloride level as summarized in figure 3. By application of the GABA_A_R antagonist bicuculline (30 μM) all post synaptic currents disappeared (not shown).

**Figure 4:**
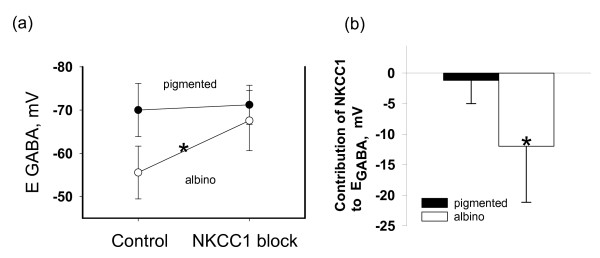
**Effects of NKCC1 blocking and calculated contribution of NKCC1 action to E_GABA _in albino and pigmented visual cortex neurons**. Effects of NKCC1 blocking (a) and calculated contribution of NKCC1 action (b) to the reversal potential of GABAAR-mediated postsynaptic currents for albino and pigmented rat visual cortex neurons. Data are presented as mean ± S.D (*p < 0.001, Mann-Whitney rank sum test).

In the presence of 10 micromolar concentration of bumetanide, a specific inhibitor of the NKCC1 cotransporter, cells with more positive values of E_GABA _than usual for neocortical neurons displayed a negative shift in E_GABA_. This negative shift in E_GABA _was observed in 7 out of 9 of albino visual cortex neurons and 1 out of 7 neurons from pigmented animals (data are summarized in fig. 4a). In line with this pharmacological effect, NKCC1 mRNA was expressed in bumetanide-sensitive neurons, but not in bumetanide-insensitive ones. The calculated contribution of NKCC1 action to the reversal potential of GABAAR-mediated postsynaptic currents for albino and pigmented rat visual cortex neurons is presented in figure 4b. Thus our pharmacological tests confirmed the link between the observed inhibitory deficit in albino visual cortex on the electrophysiological level and mRNA expression of the major chloride regulator responsible for chloride uptake in immature neural cells on the single cell molecular level.

In order to evaluate the impact of elevated intracellular chloride levels in albino visual cortex neurons on cortical network properties we conducted gramicidin perforated patch clamp analysis of both albino and pigmented layer V visual cortex neurons **without **blocking of excitation during physiological recordings. We found that the reversal potential of postsynaptic currents (combined inhibitory and excitatory) significantly differ between albino and pigmented animals, being shifted into the depolarizing direction in albinos (-49.2 +/- 1.3 mV, n = 16, in albino vs. -58.2 +/- 2.3 mV, n = 10, in pigmented animals; Additional file 1, fig. 1). One should keep in mind, however, that the observed difference may result from variable inhibitory and/or excitatory inputs to characterized cells. Surprisingly, minimal interspike intervals were significantly longer in albinos than in pigmented (Additional file 1, fig. 2): 28.2 +/- 2.9 ms(n = 16) vs. 23.2 +/- 2.6 ms(n = 10). Oscillations within and across neuronal systems are believed to serve various complex functions, such as perception, cognition, movement initiation, plasticity and memory. GABAergic mechanisms play a major role in these oscillatory patterns. Therefore, the increase in minimal interspike intervals in albino neuron may indicate a reduced possibility for high frequency neuronal coding in albino visual cortex networks. Previous findings of Barmashenko et al.[22] showed that albino rat visual cortex neurons also demonstrate a significantly lower rheobase (the current threshold to release a spike by long depolarizing pulses) and a smaller chronaxy (the minimal duration of a current pulse of twice the rheobase amplitude to release a spike) compared to pigmented animals. These data strengthen the hypothesis of a severe inhibitory deficit in albino visual cortex.

## Conclusion

In sum, our findings suggest that blocking of NKCC1 in albino visual cortex neurons will bring their chloride homeostasis to the pigmented brain level. This could improve processing in visual cortex and therefore might be beneficial for vision in albinos. So far this speculation is without supporting data and the role of NKCC1 in neuronal excitability seems controversial [24] and blocking NKCC1 in neurons without blocking in glia is not practical. In addition caution is advisable because bumetanide therapy may be controversial for retinal processing where many cell types express functional NKCC1 and their E_GABA _is either known or predicted to be positive to resting membrane potential [5,25] for normal function.

## Methods

Experiments were carried out on Long-Evans and Wistar rats of either sex of postnatal day (P) 20–40. All experiment procedures were strictly in accordance with institutional guidelines and approved by a local ethics committee. Rats were anesthetized with halothane and decapitated. The brain was taken out of the skull and immersed in ice-cold artificial cerebrospinal fluid (ACSF; 123 mM NaCl, 2.5 mM KCl, 1 mM NaH_2_PO_4_, 26 mM NaHCO_3_, 11 mM D-glucose, 1.8 mM CaCl_2_, 1.3 mM MgCl_2_, bubbled with 95% O_2 _and 5% CO_2_, pH 7.4). Visual cortex was sliced on a vibratome (MA752, Campden Instruments, Germany). Slices were stored for at least 1 h at room temperature ACSF and then relocated to a submerged recording chamber. The slices were derived from Bregma -5.8 mm to Bregma -7.5 mm [26], where the visual cortical areas 18, 17 and 18a extend 7–8 mm from the midline laterally to the temporal cortex and layer V pyramidal shaped neurons were taken into analysis. Visual cortex neurons GABA_A _receptor mediated currents were measured on different holding potentials and E_GABA _was determined from these recordings. Intracellular chloride levels of the cells were calculated from GABA_A_R mediated currents reversal potential according to the Nernst equation:

$$V_{\text{Eq}.}=\frac{RT}{zF}\ln\left(\frac{{\left[X\right]}_{\text{out}}}{{\left[X\right]}_{\text{in}}}\right)$$

where: ***V***_**Eq**_. is the equilibrium (measurable) reversible potential for a given ion, in our study it is Cl- ion; ***R ***is the universal gas constant (8.314 J.K^-1^.mol^-1^); ***T ***is the temperature in Kelvin (°K = °C + 273.15); ***z ***is the valence of the ionic species; ***F ***is the Faraday's constant (96485 C.mol^-1^); [***X***]_**out **_is the concentration of the ionic species *X *in the extracellular fluid (Cl^1-^); [***X***]_**in **_is the concentration of the ionic species *X *in the intracellular fluid (Cl^1-^).

### Gramicidin-perforated patch-clamp recordings

Techniques we used for perforated patch clamp recording have been reported in detail elsewhere [22]. The recording chamber was perfused with oxygenated room temperature ACSF, 3 ml/min. Kynurenic acid (2 mM) was added directly to the ACSF to prevent excitatory activity in the neurons tested. Gramicidin perforated patch clamp recordings were performed under visual control. Borosilicate patch electrodes (5–9 MΩ) were filled with a solution containing 130 mM K-gluconate, 0.5 mM Na-gluconate, 20 mM HEPES, 4 mM MgCl_2_, 4 mM Na_2_ATP, 0.4 mM Na_3_GTP, 0.5 mM EGTA (pH 7.2). Gramicidin (30 μg/ml, dissolved in DMSO, Sigma) was added to the solution as a membrane perforating agent. The measured membrane potentials were corrected for the junction potential of -10 mV [27]. Inhibitory postsynaptic currents (IPSCs) were evoked through a concentric bipolar electrode placed approximately 50–100 μm lateral to the recorded neuron with stimuli (20–100 μA, 50 μs duration, 0.1 Hz) in voltage clamp mode by means of a PC-501A patch clamp amplifier (Warner Institute Corporation) connected via AD/DA-converters (CED 1401+, Cambridge Electronic Design, UK) to a personal computer. Holding potentials were raised from -100 to +30 mV in 10 mV steps in every recording. Recordings underwent low-pass filtering at 3 kHz and were sampled at 10 kHz. For recording and analyzing WinWCP software (John Dempster, University of Strathclyde, Glasgow, UK) was used.

### Single cell real time PCR

cDNA synthesis and the first round of PCR was performed using OneStep RT-PCR kit (Qiagen, GmbH, Germany) as described in [10]. Briefly, cytoplasm of a cell excluding the cell nucleus was aspirated by a single mild suction. This sample was then expelled into a reaction tube which contained 5 μl of RNAse-free water with 10 units of RNAse inhibitor (Qiagen, GmbH, Germany). Harvested cytoplasm was frozen and stored at -80°C for at most 8 hours. The master mix for the reverse transcription was prepared by mixing 10 μl of 5× Qiagen OneStep RT-PCR buffer, 2 μl of 10 mM of each dNTP mix, 1 μl of 10 μM β-actin outer primers, 3 μl of 10 μM KCC2 outer primers, 3 μl of 10 μl NKCC1 outer primers, 10 units of Qiagen OneStep RT-PCR enzyme mix, 10 μl of 5× Q-solution and RNAse-free water to obtain a total volume of 40 μl. Afterwards 40 μl of the master mix were combined with 10 μl sample and the reverse transcription performed for 30 min at 50°C in a thermal cycler. After the reverse transcription step first round PCR amplification was immediately started as follows: 15 min at 95°C, followed by 40 cycles (30 s at 94°C, 30 sec at 55°C, 1 min at 72°C) in a thermal cycler. Outer primers pair's sequences for NKCC1, KCC2 and β-actin were taken from [10]. Subsequently we diluted first-round PCR products 500-fold and 1 μl of the diluted mixture was taken as a template for second round of PCR (40 cycles) in a real time PCR format using the Opticon2 detection system (Biorad, Hercules, USA) (see Additional file 1, fig. 3 for schematic of experimental procedures). For each template 10 μl SyberGreen, 0.25 μl of 10 μM inner primers and 9.75 μl of water were used (final reaction volume was 20 μl). The amplification involved a hot start activation of the polymerase at 95°C for 10 min, denaturation at 95°C for 15 s, annealing at 52°C for 30 s, extention at 72°C for 30 s in separate reactions using the inner primers pairs (β-actin, NKCC1, KCC2) for each template. These primers are specific to regions within the PCR products produced by the first round PCR amplification (nested PCR). The design of the inner primers was performed using Primer Express 2.0 software (Applied Biosystems, Foster city, USA). A possible cross-homology between NKCC1, KCC2 and β-actin sequences was excluded. The specificity of primers was further confirmed in agarose gel after amplification and by melting point analysis of the amplicons generated by Real Time PCR. Outer and inner primers sequences are presented in Additional file 1, Table 1.

### Drugs

The drugs applied were gramicidin D, bumetanide (Sigma, St. Louis, MO), kynurenic acid (KYN, an ionotropic glutamatergic receptor antagonist) and bicuculline (an ionotropic GABA_A _receptor antagonist) (Tocris Cookson, Bristol, UK). Substances were prepared as stock solutions and frozen, then added to the ACSF to reach the desired final concentration.

### Statistics

Mann-Whitney rank sum test, one way ANOVA and Chi-square tests (p < 0.001), SigmaStat software, were used to test the data for significant disparities. Numerical data are presented as mean ± S.D.

## Authors' contributions

DD curried out and designed electrophysiological and single cell real time PCR studies and draft the manuscript, AT designed inner primers sequences and participated in single cell real time PCR study and design, GZ participated in the design of single cell real time PCR study and helped to draft the manuscript, KPH conceived of the study, and participated in its design and coordination and helped to draft the manuscript.

## Supplementary Material

Additional file 1 Supplementary figure 1. Reversal potentials of postsynaptic currents in visual cortical neurons of albino and pigmented animals (excitation wasn't blocked during physiological recordings). Data are presented as mean ± S.D (*p < 0.001, one way ANOVA). Click here for file
