# Supplementary material for: Elevated intracellular chloride level in albino visual cortex neurons is mediated by Na-K-Cl co-transporter
Source: BMC Neurosci. 2008 Jun 30;9:57. doi: 10.1186/1471-2202-9-57 (PMC2453132; doi:10.1186/1471-2202-9-57)
Supplement: Additional file 1 — Supplementary figure 1. Reversal potentials of postsynaptic currents in visual cortical neurons of albino and pigmented animals (excitation wasn't blocked during physiological recordings). Data are presented as mean ± S.D (*p < 0.001, one way ANOVA). [file 1471-2202-9-57-S1.doc]

**Supplementary figure 1**

Reversal potentials of postsynaptic currents in visual cortical neurons of albino and pigmented animals (excitation wasn’t blocked during physiological recordings). Data are presented as mean ±S.D (^*^p<0.001, one way ANOVA).

**Supplementary figure 2**

Minimal interspike intervals in albino and in pigmented visual cortex neurons elicited by applying depolarizing current steps (current clamp mode, steps are 200 ms duration). Data are presented as mean ±S.D (^*^p<0.001, one way ANOVA).

## Supplementary figure 3

##

## Experimental procedure: real time singe cell PCR

## Supplementary table 1 - Outside and inner primers sequences

| Genes | ACCESSION | Type | Primer sequences | Products length (bp) |
| --- | --- | --- | --- | --- |
| ß-actin | GI:55574 | Outer | 5’-ACACGGCATTGTAACCAACT-3’  5’-CATTGCCGATAGTGATGACC-3’ | 543 |
|  |  | Inner | 5’-CTAAGGCCAACCGTGAAAAGA-3’  5’-CAACACAGCCTGGATGGCT-3’ | 86 |
| KCC2 | GI:1403708 | Outer | 5’-GATGAAGAAAGACCTGACCA-3’  5’-CTGGTTCAAGTTTTCCCACT-3’ | 523 |
|  |  | Inner | 5’-CAGCGGCTCAGAAGAACAAAG-3’  5’-TTCAAGTTTTCCCACTCCGG-3’ | 91 |
| NKCC1 | GI:3342263 | Outer | 5’-GAAAGTACTCCAACCAGAGA-3’  5’-AGCTAGAATACCAGTTGCAG-3’ | 720 |
|  |  | Inner | 5’-GTGAGAGGAGGAGGAGCATAC-3’  5’-GCGAATCCAACAACATACATAG-3’ | 125 |
